# Susceptibility testing of *Atopobium vaginae *for dequalinium chloride

**DOI:** 10.1186/1756-0500-5-151

**Published:** 2012-03-19

**Authors:** Guido Lopes dos Santos Santiago, Philipp Grob, Hans Verstraelen, Florian Waser, Mario Vaneechoutte

**Affiliations:** 1 Laboratory Bacteriology Research, Faculty Medicine & Health Sciences, University of Ghent, De Pintelaan 185, Ghent 9000, Belgium; 2 Medinova AG, Eggbuehlstrasse 14, Zurich 8052, Switzerland; 3 Department of Obstetrics and Gynaecology, Faculty of Medicine and Health Sciences, Ghent University, De Pintelaan 185, Ghent, Belgium

## Abstract

**Background:**

*Atopobium vaginae *and *Gardnerella vaginalis *are major markers for bacterial vaginosis. We aimed to determine the MIC and MBC range of the broad-spectrum anti-infective and antiseptic dequalinium chloride for 28 strains, belonging to 4 species of the genus *Atopobium, i.e. A. vaginae, A. minutum, A. rimae *and *A. parvulum*.

**Methods:**

The MIC was determined with a broth microdilution assay.

**Results:**

The MIC and MBC for *Atopobium *spp. for dequalinium chloride ranged between < 0.0625 and 2 μg/ml.

**Conclusions:**

This study demonstrated that dequalinium chloride inhibits and kills clinical isolates of *A. vaginae *at concentrations similar to those of clindamycin and lower than those of metronidazole.

## Background

Bacterial vaginosis (BV) is a polymicrobial condition whereby the lactobacilli dominated vaginal microflora is overgrown by anaerobes [1], such as *Gardnerella *vaginalis and *Atopobium vaginae *[2]. BV is the most common cause of vaginal complaints among women of childbearing age [3].

At the moment there are two recommended therapies for BV, oral metronidazole or vaginal clindamycin [4], both with one-month cure rates of 60-90% [5,6]. The recurrence rate is high, about 30-50% of the women have a relapse within 2-3 months and 50-70% within 6 to 12 months [3,5,6]. Moreover, resistance of BV-associated anaerobic bacteria to metronidazole (1%) versus 17% clindamycin resistance at baseline, and 53% clindamycin resistance after therapy has been reported [7]. In particular, it has been shown that *A. vaginae *can be metronidazole resistant [8-10].

Because both clinical and microbiological efficacy of the current antibiotic treatment of BV with metronidazole, other 5-nitro-imidazoles and clindamycin are not completely satisfactory, with a high recurrence rate of vaginosis [3,5-9], alternative treatments, such as the topical application of broad spectrum anti-infectives and antiseptics, could offer a solution to bypass possible antibiotic resistance.

One such antiseptic is dequalinium chloride, for which the broad microbicidal activity against aerobic and anaerobic bacteria as well as yeasts has been demonstrated previously [11], and for which clinical efficacy and safety in the treatment of BV and other vaginal infections has been shown [12-14].

The aim of this study was to determine the Minimal Inhibition Concentration (MIC) and Minimal Bactericidal Concentration (MBC) of DQC for strains of the genus *Atopobium*.

## Methods

### Bacterial strains

Three strains of *A. vaginae *(CCUG 44258, CCUG 44125 and CCUG 38953^T^) and one strain each of *A. minutum *(CCUG 31167), *A. rimae *(CCUG 31168) and *A. parvulum *(CCUG 32760) were obtained from the Culture Collection of the University of Göteborg, Sweden (CCUG). All other strains were clinical vaginal isolates obtained during studies between 2003 and 2010 (Table 1), and isolated after anaerobic culture (at 37°C for minimum 3 days) on Colombia agar (Becton Dickinson (BD), Erembodegem, Belgium), Schaedler agar (BD) or Tryptic Soy Agar (BD) supplemented with 5% sheep blood. All strains were identified by 16S rRNA gene sequencing. *Bacteroides fragilis *ATCC 25285^T ^was obtained from the CCUG.

**Table 1:** Values of MIC and MBC of DQC belonging to 4 species of the genus *Atopobium*

**Test No**.	Species	Strain	MIC (μg/ml)	MBC (μg/ml)
ST1	Atopobium vaginae	CCUG 44125	0.25	0.25

ST2	Atopobium rimae	CCUG 31168	1	1

ST3	Atopobium vaginae	FB101-3	0.5	0.5

ST4	Atopobium vaginae	FB106b	< 0.0625	< 0.0625

ST5	Atopobium vaginae	VMF0914COL43	< 0.0625	< 0.0625

ST6	Atopobium vaginae	VMF0914COL13	< 0.0625	< 0.0625

ST7	Atopobium vaginae	VMF0907COL23	< 0.0625	< 0.0625

ST8	Atopobium vaginae	PB2003/009-T1-4	< 0.0625	< 0.0625

ST9	Atopobium vaginae	PB2003/017-T1-2	< 0.0625	< 0.0625

ST10	Atopobium vaginae	BVS067	< 0.0625	< 0.0625

ST11	Atopobium vaginae	CCUG 44258	0.0625	0.0625

ST12	Atopobium vaginae	FB145-BA-14A	< 0.0625	< 0.0625

ST13	Atopobium vaginae	FB106B	0.0625	0.0625

ST14	Atopobium vaginae	FB158-CNA-2C	< 0.0625	< 0.0625

ST15	Atopobium vaginae	FB160-CNAB-7A	< 0.0625	< 0.0625

ST16	Atopobium vaginae	FB160-CNAB-7	< 0.0625	< 0.0625

ST17	Atopobium vaginae	FB130-CNAB-2aD	0.5	0.5

ST18	Atopobium vaginae	FB010-06	< 0.0625	< 0.0625

ST19	Atopobium vaginae	CCUG 38953^T^	< 0.0625	< 0.0625

ST20	Atopobium vaginae	FB106C	0.0625	0.0625

ST21	Atopobium vaginae	FB101-3C	< 0.0625	< 0.0625

ST22	Atopobium vaginae	BVS068	0.5	0.5

ST23	Atopobium parvulum	VMF1313W43	2	2

ST24	Atopobium parvulum	VMF1620W23	2	2

ST25	Atopobium parvulum	CCUG 32760	1	1

ST26	Atopobium minutum	CCUG 31167	2	2

ST27	Atopobium vaginae	FB101-2	< 0.0625	< 0.0625

ST28	Atopobium vaginae	PB2003/189-T1-4	< 0.0625	< 0.0625

DQC: dequalinium chloride; MBC: Minimal Bactericidal Concentration; MIC: Minimal Inhibitory Concentration

### Broth microdilution assay

A 10,240 μg/ml stock solution of DQC was prepared by dissolving 102.4 mg DQC (Analysis by Medinova AG, Zurich, Switzerland) in 10 ml HPLC water. DQC was dissolved by sonication (Labsonic 1510, B. Brauer, Melsungen, Germany) during 5 minutes at 150 W and incubation in a warm water bath (37°C) for 2 hours.

A serial dilution series ranging from 512 μg/ml to 0.0625 μg/ml DQC was used and tested in duplicate.

*Brucella *Broth (BD), supplemented with vitamin K (1 μg/ml), hemin (5 μg/ml) and laked horse blood (5%) was prepared [10]. The different *Atopobium *spp. and strains were plated onto Colombia agar (BD) and cultured for 72 hours in an anaerobic chamber (BugBox, LedTechno, Heusden-Zolder, Belgium) at 37°C. The *Brucella *Broth was pre-reduced, prior to use, for 2 hours in the anaerobic chamber at 37°C. Every cultured strain was suspended in physiological water until a 1 McFarland density (3 × 10^8 ^cfu/ml) was obtained, 1 ml of this suspension was centrifuged for 5 minutes at 7,000 *g*, and 900 μl of the supernatans was removed. In the anaerobic chamber, at 37°C, the 100 μl of remaining bacterial suspension was added to 5 ml pre-reduced *Brucella *Broth, yielding a final load of 6 × 10^6 ^cfu/ml, and homogenized.

For each six strains, two 96-well microtiterplates (Axygen, San Francisco, Ca) were used. Plate 1.1 contained concentrations of 512 μg/ml to 8 μg/ml of DQC in rows A to G and no antibiotic in row H. Plate 1.2 contained 4 μg/ml to 0.0625 μg/ml in rows A to G and no antibiotic in row H. Per strain, two columns of the two 96-well plates were filled with 100 μl of the bacterial suspension in *Brucella *broth per well.

The inoculated plates were incubated anaerobically at 37°C and read after 48 and after 72 hours.

**Figure 1:**
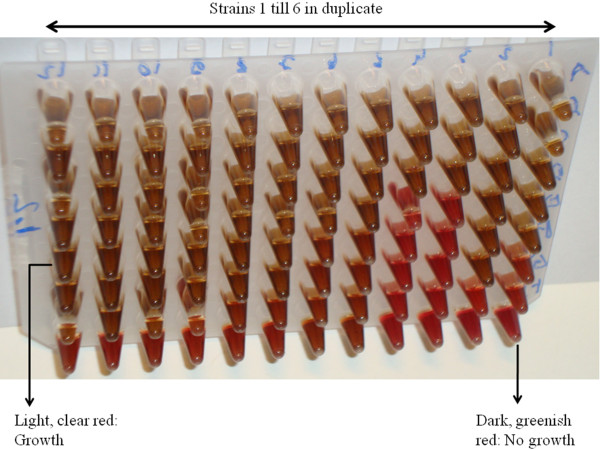
**MIC-testing of DQC in microtiterplate broth format**. MIC: Minimal Inhibitory Concentration, DQC: dequalinium chloride.

The MIC endpoint was defined as the lowest concentration of DQC that inhibited visible growth of the test isolate. Brownish, dark red wells were considered as wells without growth and clear and light red wells were considered as wells with growth (Figure 1).

For plates 1.1 and 1.2, a volume of 25 μl of each well was plated onto a Colombia agar plate (*i.e*. solid medium without DQC) to confirm the presence or absence of growth and to determine the MBC. These culture plates were incubated for 72 hours in the anaerobic chamber at 37°C.

In addition, the MICs of *A. vaginae *CCUG 44258, CCUG 44125 and CCUG 38953^T ^and *B. fragilis *ATCC 25285^T ^were tested for clindamycin and metronidazole using the broth microdilution assay under the same conditions, to validate the MIC determination approach used here.

## Results

The MIC- and MBC-values of the 28 *Atopobium *strains for DQC are shown in Table 1. The MIC and MBC range of DQC was determined as < 0.0625-2 μg/ml with an MIC90-value of 2 μg/ml.

All wells that were determined to contain bacterial growth (based on a light, clear red colour of the growth medium in the well), and that were cultured on solid medium without DQC, showed growth on the solid medium. All wells for which no growth could be observed (based on a dark, brownish red colour of the growth medium in the well) and that had been cultured on solid medium without DQC, showed no growth on solid medium. This indicates that the MIC values are similar to the MBC values, *i.e*. not only growth was inhibited at the MIC-values, as determined by growth in microtiterplate broth, but also cells were killed, as no growth could be obtained when culturing 25 μl of the broth from the wells were no growth was observed, on solid media without DQC.

The MICs of *A. vaginae *CCUG 44258, 44125 and 38953^T ^for clindamycin and metronidazole were comparable to those reported in other studies [8,15] (Table 2). The MIC of *B. fragilis *ATCC 25285^T ^for metronidazole was similar to that reported previously [15] (Table 2).

**Table 2:** Comparison of *Atopobium vaginae*isolates and *Bacteroides fragilis*type strain MIC-values (μg/ml) for clindamycin and metronidazole

	*Atopobium vaginae *CCUG 44258	*Atopobium vaginae *CCUG 44125	*Atopobium vaginae* CCUG 38953^T^	*Bacteroides fragilis* ATCC 25285^T^
Metronidazole [this study]^a^	32	128	32	1

Metronidazole [15]^b^	4	8	16	1

Metronidazole [10]^a^	NT	NT	> 32	NT

Clindamycin [this study]^a^	< 0.0625	< 0.0625	< 0.0625	< 0.0625

Clindamycin [8]^c^	< 0.016	< 0.016	< 0.016	NT

Clindamycin [16]^a^, *	NT	NT	NT	≤ 0.25 - > 16

MIC: Minimal Inhibitory Concentration; NT: Not tested

a: CLSI broth microdilution, b: Agar dilution method, c: E-test

* Range based on the MICs of 232 clinical isolates of *B. fragilis *(the type strain was not included)

## Discussion

Petersen *et al. *(2002) assessed the therapeutic efficacy of 10 mg DQC in a study population of 121 patients with various vaginal infections (bacterial vaginosis, fluor vaginalis, vulvo-vaginal candidiasis, trichomoniasis), by monitoring the clinical symptoms, the vaginal pH and the number of lactobacilli. A positive effect on the restoration of the vaginal ecosystem and good tolerability was observed, with a limited number of adverse events (5.8%) [13]. In a recent multicentre study (15 centres in 5 countries), comprising 321 women with BV, randomized to receive either DQC (n = 164) or vaginal clindamycin cream (n = 157), Weissenbacher *et al. *[14] found vaginal DQC tablets to be equally effective as vaginal clindamycin cream and to be well tolerated with no systemic safety concerns.

Together with the broad antibacterial activity of DQC against various BV-associated microorganisms, *i.e. Gardnerella vaginalis, Bacteroides *spp. and *Prevotella *spp., as shown in previous studies [11,12], our findings for *A. vaginae *DQC susceptibility add to the value of DQC as an alternative treatment for BV and vaginal infections.

The color change of the broth, which we observed when bacterial growth was absent and which was useful to determine the MIC-value, has not been previously mentioned in other publications using the same method. Possibly, it can be explained by the effect of living respectively dead bacteria on the laked horse blood in the broth.

## Conclusions

We conclude that DQC inhibits and kills clinical isolates of *A. vaginae *at concentrations similar to clindamycin and lower than metronidazole [8,15].

## Competing interests

The authors declare that they have no competing interests.

## Authors' contributions

MV and GL participated in the development of the study design and in the analysis and interpretation of the data. MV, HV, PG, FW and GL participated in the writing of the report. All authors read and approved the final manuscript.
